# Childbirth Self-Efficacy and Its Associated Factors among Pregnant Women in Arba Minch Town, Southern Ethiopia, 2023: A Cross-Sectional Study

**DOI:** 10.1155/2024/6478172

**Published:** 2024-02-15

**Authors:** Tesfahun Simon, Kassahun Fikadu, Bezawit Afework, Habtamu Alemu, Begetayinoral Kussia

**Affiliations:** Department of Midwifery, College of Medicine and Health Science, Arba Minch University, Arba Minch, Ethiopia

## Abstract

**Background:**

Childbirth self-efficacy is a pregnant women's perception of their ability to cope with labor stress. Low childbirth self-efficacy is linked to pain intolerance and poor labor progression, which increase the possibility of operative delivery. However, Ethiopia has limited data. So, the aim of this study was to assess childbirth self-efficacy and its factors among pregnant women attending antenatal care in public health facilities in Arba Minch town, Southern Ethiopia, in 2023.

**Objective:**

To assess childbirth self-efficacy and associated factors among pregnant women attending antenatal care in public health facilities in Arba Minch town, Southern Ethiopia, in 2023.

**Methods:**

An institution-based cross-sectional study was carried out among 416 women from January 1 to January 30, 2023. A systematic random sampling technique was employed. Data were collected by KoboToolbox through face-to-face interviews using a structured and pretested questionnaire. Modified short-form childbirth self-efficacy inventory was used to score self-efficacy. The Statistical Package for Social Sciences, version 27, was used for data management and analysis. Descriptive statistics were calculated for each variable, and a logistic model was used. Statistical significance was determined at a *p* value of less than 0.05 and 95% confidence level.

**Results:**

A total of 416 pregnant women participated in the study. Two hundred twenty-eight (54.8%) of the pregnant women had low childbirth self-efficacy. Age group in ≤24 years (AOR = 3.80, 95% CI: 1.82-8), primigravida (AOR = 1.51, 95% CI: 1.10-2.86), unplanned pregnancy (AOR = 1.67, 95% CI: 1.02-2.70), poor social support (AOR = 2.17, 95% CI: 1.09-4.30), having anxiety (AOR = 1.30, 95% CI: 1.10-3.64), having poor knowledge of childbirth (AOR = 2.21, 95% CI: 2.09-5.39), and severe fear of childbirth (AOR = 6.40, 95% CI: 2.60-9.80) were statistically significant with low childbirth self-efficacy.

**Conclusions:**

The magnitude of low childbirth self-efficacy was high in the study area. Being primigravida, unplanned pregnancy, age ≤ 24 years, severe fear of childbirth, anxiety, poor social support, and poor knowledge were significantly associated with low childbirth self-efficacy. Therefore, giving special attention to these factors during antenatal care would be important.

## 1. Introduction

Childbirth is a normal biological process that involves physiological, emotional, psychological, and physical changes in women's lives that can influence the sociocultural context and the morbidity and mortality of the mother and child [1, 2]. Self-efficacy is primarily defined as both the belief that individuals can successfully complete a task (self-efficacy expectancy) and the achievement of an estimated specific outcome (outcome expectancy) [3, 4]. Childbirth self-efficacy refers to a mother's belief that she can have a normal vaginal birth with minimal intervention, as well as a strong ability to cope with labor pain and have control over adversity in the event of maternal, fetal, or newborn complications [5, 6].

Regarding the ways in which stronger self-efficacy would be developed, Bandura asserts that it can happen in four ways. The first way is through the vicarious experiences provided by social models (like birth stories from other women, childbirth education videos, and media). Social (verbal) persuasions are a second way of strengthening people's beliefs; receiving positive verbal feedback while undertaking a complex task persuades a person to believe that they have the skills and capabilities to succeed. The third one is that the emotional and psychological well-being of a person can influence how they feel about their personal abilities in a particular situation, while control over negative emotion is the last one [4].

According to the World Health Organization, childbirth self-efficacy may promote a positive childbirth experience, which is an important aspect of intrapartum care. A report from high-income countries indicates that about 52% of pregnant women had low childbirth self-efficacy [1]. In addition, studies in some Asian countries showed higher low levels of childbirth self-efficacy ranging from 63.7% to 82% [5, 7]. As the evidence showed, women with low childbirth self-efficacy are more likely to experience severe pain during labor and delivery, use anesthesia during labor, and have a longer labor period, which could have a negative effect on the well-being and development of women and also newborn [8]. Pregnant women's low self-efficacy also affects the mode of delivery, in which women's childbirth fear increases the rate of cesarean section due to an exaggerated perception of the risk of vaginal delivery [9]. Moreover, low childbirth self-efficacy can affect maternal well-being and mother-infant bonding; it can lead to a condition of psychological distress as well as to severe forms of disease like postpartum depression [10].

According to different literature, factors that influence pregnant women's childbirth self-efficacy during childbirth are age, education level, gravidity, parity, pregnancy planning, previous cesarean delivery, fear of childbirth, social support, and anxiety [7, 11–15]. Moreover, available studies are conducted in high-income countries, and little is known according to problems in low-income countries. Studies were scarcely investigated on sociodemographic, obstetric, and psychometric factors such as occupational status, gestational age, prenatal depression, and knowledge of childbirth. Somehow, the current study added some sociodemographic, obstetric, and psychometric characteristics of women affect their childbirth self-efficacy perception in both positively and negatively. As far as our knowledge is concerned, no study was conducted regarding to childbirth self-efficacy in study area. Given the diverse geographical context and sociocultural differences, studying the status of perceived childbirth self-efficacy is helpful for understanding how women cope with labor and their birth experience. Thus, the current study was done to assess the childbirth self-efficacy and associated factors among pregnant women attending antenatal care in public health facilities in Arba Minch town, Southern Ethiopia.

## 2. Methods and Materials

### 2.1. Study Setting, Period, Design, and Population

An institution-based cross-sectional study was applied from January 1 to January 30, 2023, in the public health facilities in Arba Minch town. Gamo Zone's administrative center is Arba Minch town in Southern Ethiopia. It lies 495 kilometers south of Ethiopia's capital city of Addis Ababa. In the town, antenatal care (ANC) services were offered to pregnant women by four government health care facilities (one general hospital, one primary hospital, and two health centers) [16]. The source population was all pregnant women who attended antenatal care in public health facilities in Arba Minch town, and pregnant women who attended antenatal care in public health facilities in Arba Minch town during the data collection period were the study population.

### 2.2. Inclusion and Exclusion Criteria

All pregnant women were eligible for study. Women who had medical issues or faced pregnancy-related complications that could impact their capacity to provide consent and respond to inquiries were not included in the study.

### 2.3. Sample Size Determination and Sampling Technique

Using a single population proportion formula, the sample size was calculated. Given that the prevalence of low childbirth self-efficacy was unknown and that no prior research had been conducted in nations with comparable socioeconomic characteristics, an estimate of 50% was applied in this study, with a margin of error of 5% and a 95% confidence level of certainty. Based on this information and a 10% nonresponse rate, the final sample size was *N* = 422.

**Figure 1:**
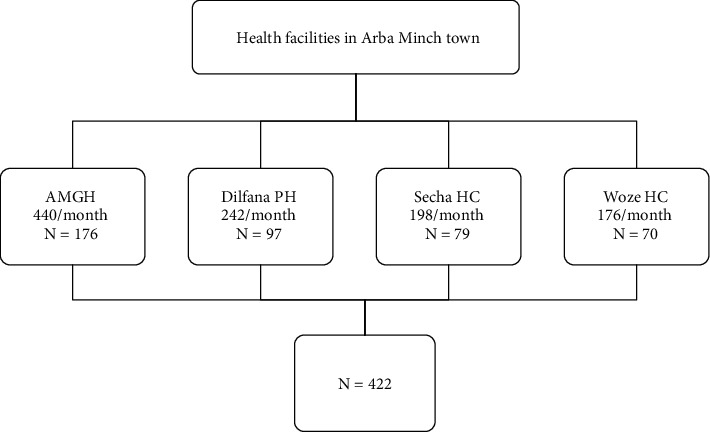
Schematic presentation of the sampling procedure to assess childbirth self-efficacy and associated factors among pregnant women attended antenatal care in public health facilities in Arba Minch town, Southern Ethiopia, in 2023.

According to the annual report for ANC services provided by the public health facilities in Arba Minch town, daily antenatal care visits by pregnant women average around 20 at Arba Minch General Hospital, 11 at Dilfana Primary Hospital, 9 at Secha Health Center, and 8 at Woze Health Center. The interval was computed based on the anticipated number of pregnant women visiting the prenatal care center throughout the data collecting period. Based on the flow of the pregnant women through the health care facility, the total sample size was proportionally allocated to the health facility; finally, a sample from each health facility was reached by using the systematic sampling technique, which involved every two pregnant women available at the ANC clinics during the data collection period for each facility. To minimize systematic sampling bias, we clearly outlined the target population and enumerated the individuals from whom the sample was selected. The first participant was selected by the lottery method (1 out of 2 women) (Figure 1).

### 2.4. Operational Definition

*Childbirth self-efficacy*: a woman's belief in her ability to cope with labor and delivery. Women who scored mean or above on the 30 childbirth self-efficacy inventory items were considered to high childbirth self-efficacy [17].

*Perceived quality of care*: women's view of service received. It measured women's responses to six questions regarding aspects of the quality of their antenatal care, and a good perceived quality was having a mean or above average score [18].

*Fear of childbirth*: feelings of uncertainty and anxiousness before, during, and after the labor and birth. In this study, fear of childbirth was assessed on 33 W-DEQ items with a sum score of 38 for low degree fear, 38–65.9 for moderate degree fear, 66–84.9 for high degree fear, and 85 for severe degree fear of childbirth [19].

*Antenatal depression*: pregnant women experience negative emotional changes for 3–4 days. In this study, depression was measured by nine items, and having a score of more than five was taken as antenatal depression [20].

*Antenatal anxiety*: excessive worry and fear of labor and birth. In this study, seven antenatal anxiety items were evaluated, and pregnant women with a score of more than five were considered anxious [20].

*Knowledge of childbirth*: knowledge of women on natural birth. It was assessed based on the women's responses to seven knowledge questions; thus, scores of mean or above were considered as knowledgeable about childbirth [12].

*Social support*: a categorical variable that is assessed using the three questions from Oslo items. Women with scores of 3 to 8 are considered to have poor social support, 9 to 11 to have moderate support, and 12 to 14 to have strong support [11].

### 2.5. Data Collection

Data were collected using structured and pretested questionnaires including the modified childbirth self-efficacy inventory (CBSEI), Wijma Delivery Expectation and Experience Questionnaire (W-DEQ) for fear of childbirth, Oslo social support, generalized anxiety disorder 7 items, Patient Health Questionnaire (PHQ) items, and women's perceived quality of care items through face-to-face interview technique. Six BSc midwives participated in the data collection and two MSc on clinical midwifery supervision after taking training for two days. Modified short-form CBSEI has been designed to measure childbirth self-efficacy with a 30-item rating scale of 5 Likert scales as a response format, ranging from “not at all sure” to “completely sure.” The items' total scores, which can range from 30 to 150, might be high or low; a high score denotes a high level of childbirth self-efficacy. Based on the total scale's Cronbach's alpha score of 0.94, it had a reliable internal consistency.

Childbirth self-efficacy was operationalized as women who scored mean or above on the 30 childbirth self-efficacy inventory items were considered to high childbirth self-efficacy [17]. In this study, fear of childbirth was assessed on 33 W-DEQ items with a sum score of ≤38 for low degree fear, 38–65.9 for moderate degree fear, 66–84.9 for high degree fear, and ≥85 for severe degree fear of childbirth [19]. Social support is a categorical variable that is assessed using three questions from Oslo items. Women with scores of 3 to 8 are considered to have poor social support, 9 to 11 to have moderate support, and 12 to 14 to have strong support [21]. Depression was measured by nine items, and having a score of more than five was taken as antenatal depression [20]. Seven antenatal anxiety items were evaluated, and pregnant women with a score of more than five were considered anxious [20]. Perceived quality of care is women's view of the service received. It measured women's responses to six questions regarding aspects of the quality of their antenatal care, and a good perceived quality was having a mean or above average score [18]. Knowledge of childbirth was assessed based on the women's responses to seven knowledge questions; thus, scores of mean or above were considered as knowledgeable about childbirth [12].

### 2.6. Data Quality Assurance and Analysis

The tool was translated from the English language to Amharic and retranslated to the English version before data collection to ensure consistency. A pretest was done with 20 pregnant women at another health facility near the study area. The collected data was downloaded from KoboToolbox and exported to Statistical Package for Social Sciences (SPSS) version 27 for further analysis. A test of normality was checked to select the appropriate statistical summary measure. Descriptive statistical analyses were computed and presented in text table. The association between an outcome variable and each independent variable was seen separately in a binary logistic regression model. Binary logistic regression is a statistical technique employed for modeling the association between a binary dependent variable (typically coded as 0 and 1, representing two possible outcomes) and one or more independent variables. This method extends the principles of simple logistic regression, where the prediction of a binary outcome is based on a sole predictor variable [22]. Variables with a *p* value of less than 0.25 in bivariate analyses were entered for multivariable analyses. Multicollinearity was checked to see the linear correlation among the independent variables by correlation coefficient and variance inflation factors. The degree of association between an outcome variable and independent variables was determined using an adjusted odds ratio along with a 95% CI and a *p* value less than 0.05. Hosmer-Lemeshow's model goodness-of-fit test was done, and it was 0.89.

## 3. Results

### 3.1. Sociodemographic Characteristics of the Study Participants

A total of 416 pregnant women participated in the study, with a response rate of 98.6%. The mean age of the respondents was 27 ± 6 years, and the majority of 281 (67.5%) were in the age groups between 25 and 34 years (Table 1).

### 3.2. Obstetric Characteristics of the Study Participants

From the obstetrical characteristics of the respondents, 249 (59.9%) were multigravidas. About 246 (59.1%) were planned pregnancy (Table 2).

### 3.3. Psychometric Characteristics of the Participants

Among participants, 33 (7.9%) of pregnant women had low degree fear (sum score of <38), 229 (55%) had moderate degree fear (sum score of 38-65.9), about 92 (21.2%) pregnant women had high degree fear (sum score of 66-84), and 62 (14.9%) of pregnant women had severe degree fear of child (sum score of ≥85). Concerning for antenatal depression, 364 (87.5%) of pregnant women had no antenatal depression, whereas 304 (73.1%) of pregnant women had no antenatal anxiety.

### 3.4. Other Factors

Concerning the knowledge about childbirth, 235 (56.5%) of the participants had good knowledge of childbirth. Regarding social support, 72 (17.3%), 215 (51.7%), and 129 (31.3%) of pregnant women had poor, moderate, and strong social support, respectively. Regarding the women's perceived quality of care, 257 (61.8%) were good and 159 (38.2%) were poor.

### 3.5. Magnitude of Childbirth Self-Efficacy of Pregnant Women

**Figure 2:**
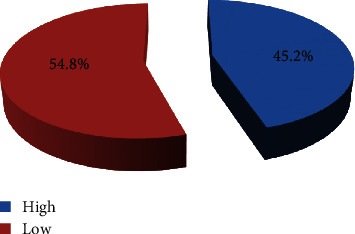
Schematic picture of childbirth self-efficacy of pregnant women attending antenatal care in Arba Minch town in Southern Ethiopia, 2023.

In this study, 228 (54.8%) of pregnant women had low childbirth self-efficacy and 188 (45.2%) of pregnant women had high childbirth self-efficacy (Figure 2).

### 3.6. Factors Associated with Childbirth Self-Efficacy

As determining factors of low childbirth self-efficacy were likely to be important, we have executed bivariate and multivariable analyses for low childbirth self-efficacy. Accordingly, a total of twelve variables were candidates for multivariable analysis. In multivariable logistic regression analysis, age group in ≤24 years, primigravida, unplanned pregnancy, poor social support, anxiety, poor knowledge of childbirth, and a severe degree of fear of childbirth were factors associated with low childbirth self-efficacy (Table 3).

## 4. Discussion

In this study, self-efficacy of childbirth among pregnant women attending antenatal care services in public health facilities in Arba Minch town was studied. More than half of pregnant women had low childbirth self-efficacy. This study revealed that about 54.8% (95% CI: 50.00%-60.10%) of the study participants had low childbirth self-efficacy. The finding in this study is in line with the results of the Swedish study (52%) [1].

The results revealed that low childbirth self-efficacy in this study is lower than the findings from Iraq (82%) and Indonesia (63.7%) [7, 12]. These discrepancies may result from variations in the sample size, study population, sampling techniques, and differences in health institution structure and in tools used to measure the level of childbirth self-efficacy, as well as from cultural and attitudinal variations.

According to the current study, a pregnant woman's age has an effect on how confident she feels about childbirth. Compared to their counterparts, women in the age group of ≤24 years were 3.8 times more likely to have low childbirth self-efficacy. The research done in Turkey, China, and Indonesia gives support to this [12, 23, 24]. The possibility is that as the pregnant women do not become advanced age, they may not learn to adapt and gain more information about childbirth, to improve their confidence in their ability to cope with it.

Compared to multigravida women, primigravida women were 1.51 times more likely to have low childbirth self-efficacy. The research done in Australia and Singapore gives support to this [7, 17]. The possible justification may be due to physiological adaptation and being well prepared for childbirth. When compared with their counterparts, pregnant women who had poor knowledge about childbirth were 2.2 times more likely to have low levels of childbirth self-efficacy. This is in line with the findings of studies conducted in Indonesia [12]. This could be because pregnant women may have more knowledge about childbirth due to the mass media, professional guidance and counseling, and other sources that make them feel more confident about childbirth.

Pregnant women with unplanned pregnancies were 1.67 times more likely to have lower childbirth self-efficacy than those with planned pregnancies. This finding is similar to a Turkish research that found that women who had unplanned pregnancies experienced lower levels of childbirth self-efficacy [12]. The reason might be that women who had unplanned pregnancies have increased stress and are less prepared physically, mentally, socially, and economically, which led to the mother less confident in their labor and delivery.

Compared to their counterparts, pregnant women with severe levels of childbirth fear were 6.4 times more likely to have low levels of childbirth self-efficacy. Studies from Sweden and the United States give support to this [24, 25]. This might be due to the lack of awareness about childbirth provided by health services, and an undesirable pregnancy might also increase fear.

Moreover, social support was also an important factor in this study. When compared to pregnant women who had strong social support, those with poor social support were 2.1 times more likely to have low childbirth self-efficacy. Studies done in Turkey confirm this finding [14]. The strong support from family, neighbors, and other stakeholders may enhance the woman's perception that childbirth is a physiological and natural process, leading to improving psychological well-being and raising childbirth self-efficacy, which is one reason that might be offered.

Compared to their counterparts, women who did experience anxiety had low levels of self-efficacy of childbirth. The results of this investigation agreed with those of studies done in Sweden, Iran, and Arak [1, 26, 27]. This may be due to the fact that women with low levels of self-efficacy for childbirth may have limited capacity to handle labor pain. In addition, women felt incapable of the activities or effort needed to manage labor, which increases excessive fear and stress.

This study is the first of its kind and has highlighted that some important aspects of childbirth self-efficacy in Ethiopia women may be taken as the strength of the study. However, the main limitation of this study was as the study participants were recruited from only those who come to health facilities, it is difficult to generalize the findings to the setting outside and limited number of related literature.

## 5. Conclusions and Recommendations

This finding showed the high magnitude of low childbirth self-efficacy in pregnant women in this study area. Age group in ≤24 years, primigravida, unplanned pregnancy, poor social support, anxiety, poor knowledge of childbirth, and severe degree of fear of childbirth were factors associated with low childbirth self-efficacy. Therefore, giving special attention to these factors during antenatal care would be important. Early detection of pregnant women who experience anxiety and lack of social support allows for specific attention and cognitive behavioral therapy. Prenatal education and encouragement are needed regarding childbirth to increase primigravida women's belief in their own capability to control and cope with labor stress. Health care givers should give appropriate information and services regarding to how to prevent unplanned. For subsequent researchers, it is important to include wide areas and mixed methods used to explore the factors and levels of childbirth self-efficacy.

## Figures and Tables

**Table 1:** Pregnant women's sociodemographic characteristics attending antenatal care in public health facilities of Arba Minch town, Southern Ethiopia, 2023 (*N* = 416).

Variables	Category	No.	%
Age of women	≤24	90	21.6
	25-34	281	67.5
	≥35	45	10.8

Marital status	Married	413	99.3
	Unmarried	3	0.7

Women's educational status	Unable to read and write	22	5.3
	Able to read and write	128	30.8
	Primary education	126	30.3
	Secondary education	70	16.8
	College and above	70	16.8

Women's occupation	Housewife	271	65.1
	Employed	49	11.8
	Merchant	66	15.9
	Student	30	7.2

Husband educational status	Unable to read and write	18	4.3
	Able to read and write	138	33.2
	Primary education	72	17.3
	Secondary education	67	16.1
	College and above	121	29.1

Husband occupation	Government employee	158	38.0
	Merchant	98	23.6
	Student	26	6.3
	Daily labors	134	32.2

Number of household members	1-5	346	83.2
	Above 5	70	16.8

Household monthly income	≤2500	99	23.8
	2501-3999	117	28.1
	≥4000	200	48.1

**Table 2:** Pregnant women's obstetric characteristics attending antenatal care in public health facilities in Arba Minch town, Southern Ethiopia, 2023 (*N* = 416).

Variables	Category	Frequency	%
Number of pregnancies	Primigravida	167	40.1
	Multigravida	249	59.9
	Total	416	100.0

Number of live births (*N* = 249)	Primiparous	133	53.4
	Multiparous	116	46.6
	Total	249	100

Gestational age in week categories	28-36	349	83.9
	37-42	67	16.1
	Total	416	100.0

Current pregnancy problems	Yes	14	3.4
	No	402	96.6
	Total	416	100.0

Previous C/S (*N* = 249)	Yes	55	22
	No	194	78
	Total	249	100

Planned pregnancy	Yes	246	59.1
	No	170	40.9
	Total	416	100.0

Husband involvement during antenatal visit	Never	110	26.4
	Once	214	51.4
	More	92	21.1
	Total	416	100.0

**Table 3:** Multivariable logistic regression analysis of childbirth self-efficacy among pregnant women in Arba Minch town, Southern Ethiopia, 2023 (*N* = 416).

Variables		Childbirth self-efficacy		COR (95% CI)	AOR (95% CI)	*p* value
		Low	High			
Gravidity	Multigravida	125 (54.8%)	124 (66%)	1		
	Primigravida	103 (45.2%)	64 (34.0%)	1.59 (1.07-2.37)	1.51 (1.10-2.86)	0.01^∗^

Planned pregnancy	Yes	121 (53.1%)	125 (66.5%)	1		
	No	107 (46.9%)	63 (33.5%)	1.75 (1.17-2.60)	1.67 (1.02-2.73)	0.03^∗^

Knowledge of childbirth	Good	109 (47.8%)	126 (67%)	1		
	Poor	119 (52.2%)	62 (33%)	2.22 (1.48-3.31)	2.21 (2.09-5.39)	0.001^∗^

Fear of childbirth	Low degree	10 (4.4%)	23 (12.2%)	1		
	Moderate	113 (49.6%)	116 (61.7%)	2.24 (1.02-4.92)	2.25 (1.10-6.58)	0.030^∗^
	High	59 (25.9%)	33 (17.6%)	4.1 (1.74-9.67)	3.97 (1.52-10.3)	0.005^∗^
	Sever	46 (20.2%)	16 (8.5%)	6.6 (2.59-10.84)	6.4 (2.66-9.80)	0.001^∗^

Social support	Strong	61 (26.8%)	68 (36.2%)	1		
	Moderate	119 (52.2%)	95 (50.5%)	1.57 (1.01-2.44)	1.22 (0.73-2.01)	0.440
	Poor	48 (21.1%)	25 (13.3%)	2.60 (1.42-4.77)	2.17 (1.09-4.29)	0.026^∗^

Age of women	≥35	17 (7.5%)	28 (14.9%)	1		
	25-34	148 (64.9%)	133 (70.7%)	1.80 (0.96-3.49)	1.62 (0.78-3.35)	0.19
	≤24	63 (27.6%)	27 (14.4%)	3.84 (1.81-8.15)	3.8 (1.82-10.0)	0.001^∗^

Anxiety	No anxiety	163 (71.5%)	160 (85.1%)	1	1	
	Having anxiety	65 (28.5%)	28 (14.9%)	1.32 (0.85-2.05)	1.30 (1.10-3.64)	0.021^∗^

Key: 1 = reference. ^∗^Variable with *p* values < 0.05.

## Data Availability

Datasets used or analyzed during the current study are available from the corresponding author upon reasonable request.

## Abbreviations

ANC: Antenatal care
CBSE: Child birth self-efficacy
CBSEI: Child birth self-efficacy inventory
HC: Health center
PHQ: Patient Health Questionnaire
W-DEQ: Wijma Delivery Expectation/Experience Questionnaire.
